# Arrhythmic Mitral Valve Prolapse as a Regional Stretch-Induced Cardiomyopathy with Fibro-Inflammatory Signature: A Conceptual Review

**DOI:** 10.3390/jcdd13090437

**Published:** 2026-09-05

**Authors:** Mili Dave, Shaaf Ahmad, Kirkwood Adams, Adam Akerman, Jinny Ha, Ross J. Simpson, Faisal F. Syed

**Affiliations:** Department of Medicine, Chapel Hill School of Medicine, University of North Carolina, Chapel Hill, NC 27599, USA; shaaf@email.unc.edu (S.A.); kfa@med.unc.edu (K.A.); adam_akerman@med.unc.edu (A.A.); jinny_ha@med.unc.edu (J.H.); ross_simpson@med.unc.edu (R.J.S.J.);

**Keywords:** mitral valve prolapse, arrhythmic mitral valve prolapse, mitral annular disjunction, myocardial fibrosis, substrate-modifying therapy

## Abstract

Mitral valve prolapse (MVP) affects 1–3% of the general population and is largely considered benign. However, a small subset of MVP patients develops complex ventricular arrhythmias (VAs) and sudden cardiac death, including patients with nonsignificant mitral regurgitation (MR). This subgroup of arrhythmic MVP (aMVP) is variably associated with features such as mitral annular disjunction, bileaflet myxomatous prolapse, and regional myocardial fibrosis, with arrhythmic risk disproportionate to hemodynamic burden. The EHRA defines aMVP as MVP with complex VAs (frequent premature ventricular contractions, non-sustained or sustained ventricular tachycardia, ventricular fibrillation, or aborted sudden cardiac arrest) in the absence of another defined arrhythmic substrate. This narrative review reframes aMVP as a regional, stretch-induced cardiomyopathy with a distinct fibro-inflammatory signature. We further characterize a two-hit pathogenesis in which abnormal valvular mechanics create regional myocardial stress, while patient-specific vulnerability shapes variable fibro-inflammatory, fibrotic, and electromechanical responses. This narrative review synthesizes contemporary evidence across multimodal cardiac imaging, electrophysiology, surgical outcomes, mechanotransduction biology, and pharmacology, emphasizing a modifiable fibro-inflammatory trajectory underlying the intermediate risk aMVP phenotype and supporting a clinical shift from device-based rescue to substrate-directed prevention. Expanding on this model, we evaluate substrate-modifying pharmacotherapies for mechanistic fit, human cardiac evidence, and trial feasibility. Among these, mineralocorticoid receptor antagonists (MRAs) and sodium–glucose cotransporter 2 (SGLT2) inhibitors demonstrate the strongest convergence of antifibrotic potential and trial readiness.

## 1. Introduction

Mitral valve prolapse (MVP), estimated to affect 1–3% of the general population, has historically been considered benign. However, current epidemiological data reveal a troubling paradox: a subset of apparently “low-risk” MVP patients experience sudden cardiac death at an estimated annual risk of 0.2–1.9% [1,2,3]. Demographically, these patients are often young and predominantly female, with no or minimal mitral regurgitation (MR) and preserved left ventricular function [4]. Malignant ventricular arrhythmias (VAs) in such patients without overt evidence of structural heart disease encourage a re-evaluation of MVP beyond recognition of risk factors to explain how a seemingly benign valvular abnormality might precipitate a malignant arrhythmic substrate in a subset of patients.

The current clinical management of arrhythmic MVP (aMVP) remains reactive and limited, with patients undergoing surveillance imaging and rhythm monitoring, catheter-based ablation for arrhythmic triggers when clinically evident, and ICD placement after near-fatal events [5,6,7]. Complex and frequent ventricular ectopy (cfVE) originating from the fascicular and papillary muscle foci is commonly encountered among MVP patients, both with and without sudden cardiac death (SCD). While beta blockade currently remains first-line for symptomatic MVP-related cfVE, medical management has not been conclusively shown to improve survival [8], and evidence for substrate-modifying pharmacotherapy remains additionally limited [9]. The absence of a consolidated approach reflects a gap in the current mechanistic understanding of how valvular pathology that translates into myocardial electrical instability.

The pathogenesis of aMVP is likely multifactorial: the prolapse of mitral leaflets, particularly in the setting of mitral annular disjunction (MAD) and bileaflet myxomatous involvement, exerts repetitive mechanical traction on the papillary muscles and inferobasal left ventricular wall [10]. This repetitive mechanical stretch is hypothesized to initiate a cascade of cellular responses including myofibroblast transformation and extracellular matrix expansion via profibrotic cytokines and inflammatory cells, ultimately setting the foundation for regional myocardial fibrosis [11,12]. To better conceptualize the diverse factors implicated in aMVP pathogenesis, and the putative interventions that might prevent it, we propose framing aMVP as a stretch-induced cardiomyopathy: a process in which repetitive stretch-induced injury from abnormal cardiac biomechanics results in myocardial injury, inflammation, fibrosis, myocardial dysfunction, and arrhythmia. We explore how mechanically induced inflammation may be coupled to fibrosis, serving as both substrate and modulator of ventricular arrhythmogenesis. This framework builds on hybrid positron emission tomography/cardiac magnetic resonance imaging (PET/CMR) evidence showing that regional ^18^F-FDG myocardial uptake colocalizes with late gadolinium enhancement (LGE) in patients with bileaflet MVP and ventricular arrhythmia [1,10,13].

Characterizing aMVP as a stretch-induced cardiomyopathy with fibro-inflammatory remodeling encourages expansion beyond the current limited therapeutic approach to consider substrate-modifying pharmacotherapy for secondary prevention and arrhythmic risk modification. We introduce potentially repurposable therapeutic targets on four primary axes: fibrosis (interstitial and replacement), innate immunity and inflammasome signaling (e.g., connective tissue signaling modulators), mechanosensing and neurohormonal activation, and calcium modulation that transforms a remodeled ventricle into an arrhythmogenic substrate. The progressive profibrotic myocardial changes observed in both murine models and aMVP patient biopsies [14] provide a further mechanistic rationale for early intervention on the above axes.

We synthesize contemporary evidence on the pathophysiology, risk stratification, and clinical management of aMVP, emphasizing mechanistic links between abnormal valvular mechanics, myocardial remodeling, and ventricular arrhythmogenesis. By reframing aMVP not simply as a valvular disease but as a regional cardiomyopathic process, we aim to identify research priorities for mechanism-based prevention. To our knowledge, this is the first synthesis to integrate aMVP-specific imaging, surgical, genetic, and pathologic data with mechanotransduction biology and repurposable pharmacotherapy into a valve–ventricle prevention framework. The integration of mechanistically adjacent evidence reflects the current state of the field and is made explicit throughout the review.

## 2. Part I: Clinical Phenotype and Mechanistic Framework

### 2.1. Arrhythmic MVP: The Clinical Phenotype

Operationally, aMVP is defined as MVP with one or more of: cfVE, sustained or non-sustained ventricular tachycardia, ventricular fibrillation, or aborted sudden cardiac arrest, in the absence of another defined arrhythmic substrate such as primary cardiomyopathy, channelopathy, active ischemia, or ventricular scar of another etiology [4,15,16]. Although the tetrad of women with bileaflet myxomatous morphology without hemodynamic burden, cfVE, and inferolateral T-wave abnormalities is a repeatedly described core [17,18,19], no component of this tetrad is obligate. In an international registry of 148 patients with arrhythmogenic MVP and sudden cardiac arrest or sustained VT/VF, men accounted for 32%, prolapse was confined to a single leaflet in 27%, inferolateral T-wave inversion was absent in 40%, and LVEF was below 50% in 13% [20]. Observations of ventricular fibrosis patterns in aMVP have enhanced our understanding of how these features relate to arrhythmogenesis. In the meta-analysis by Pistelli et al. [15], LGE-defined fibrosis on cardiac MRI was the strongest discriminator between arrhythmic and non-arrhythmic forms of MVP (OR 16.67; *p* = 0.005), correlating with sites of origin of ectopy from the peri-mitral annular regions and LV papillary muscles, as well as regional distribution of T-wave inversions. Similarly, interstitial fibrosis quantified by global ECV% is associated with aMVP in women without severe MR or MAD [21]. In a recent prospective CMR cohort of 550 patients [22], female sex (HR 3.33; 95% CI 1.72–6.44; *p* < 0.001) and LGE presence (HR 3.17; 95% CI 1.74–5.77; *p* < 0.001) were significantly associated with arrhythmic events. Women with fibrosis represented the highest-risk subgroup, supporting sex-specific characterization in the phenotype definition.

### 2.2. Imaging Markers as Mechanical Proxies

#### 2.2.1. Mitral Annular Disjunction

Imaging in aMVP serves as a tool to quantify maladapted mechanical forces transmitted to the myocardium. MAD, frequently present in aMVP patients, introduces repetitive mechanical distortion of the papillary muscles and inferobasal left ventricular wall through systolic billowing of the mitral valve [10,18], stimulating apoptotic pathways and ultimately inducing regional hypertrophy and fibrosis [10,19]. Individuals with MVP and MAD are more likely to have cfVE and worse outcomes than those without MAD [16,23]. As shown in a recent multimodality imaging study by King et al. [24], reduction in cardiac output (CO) and cardiac index (CI) is associated with MAD severity. Any MAD (≥2 mm) was associated with lower CI versus MVP-only (*p* = 0.03), with MAD ≥ 5 mm and ≥ 10 mm demonstrating significant reductions in global cardiac performance: CI (*p* = 0.042 and *p* = 0.027) and CO (*p* = 0.040 and *p* = 0.039). Although greater severity of MAD is associated with increased cfVE [25,26], the presence of MAD is neither requisite nor sufficient for arrhythmogenesis. MVP patients without MAD may have malignant VAs, and many with MAD remain free of overt arrhythmia [27,28]. These findings suggest additional arrhythmogenic substrate-mediating factors that may be present in MVP, spanning both abnormal regional mechanics and electromechanical heterogeneity. Each is more prominent when MAD is present and may be amplified by it, yet each has been described in MVP without MAD; the relationship is therefore best understood as coupled rather than causal in either direction. In a formal mediation analysis by Marra et al., curling and MAD exerted their arrhythmic effect partly through myocardial fibrosis, while curling also showed a direct, fibrosis-independent effect, highlighting association and mechanistic plausibility, rather than proven causation [10,27,28].

#### 2.2.2. Abnormal Regional Mechanics

Beyond MAD, MVP imposes maladaptive mechanical loading that is visible as abnormal regional kinematics on multiple imaging modalities. Repetitive systolic “curling” of the basal inferolateral myocardium reflects exaggerated annular and basal wall motion driven by leaflet billowing. Spiked or multi-peaked configurations of lateral annular tissue Doppler velocities, most familiar as the Pickelhaube sign, a sharp lateral mitral annular systolic velocity ≥16 cm/s, capture the same hyperdynamic basal motion in a different modality. Speckle-tracking adds a third view: the double-peak strain pattern, reflecting late-systolic inferobasal lengthening before and after end-systole, shows a significant independent association with arrhythmia even after adjustment for fibrosis and prior arrhythmic events (HR 3.12; 95% CI 1.25–7.78; *p* = 0.014) [28,29]. In a prospective observational study by Nagata et al. [29], this pattern was absent in patients with inferolateral fibrosis from etiologies unrelated to MVP, indicating that it captures MVP-specific mechanical distortion rather than fibrosis itself. Consistent findings also emerge on CMR feature-tracking: among 52 patients with bileaflet MVP, Gatti et al. found that reduced global longitudinal strain and abnormal post-systolic strain rate identified patients with sustained ventricular tachycardia or fibrillation, and diastolic strain rate abnormalities distinguished those with papillary muscle LGE. This reinforces regional deformation as a possible modality-independent marker of arrhythmic substrate [30]. Each of these markers is more prominent in the presence of MAD but has been described in MVP without MAD, supporting the framing of MAD and abnormal regional mechanics as coupled but partially separable substrates.

#### 2.2.3. Electromechanical Heterogeneity

Whereas regional mechanical markers describe how the ventricle moves abnormally, electromechanical heterogeneity describes the tissue-level substrate that translates abnormal loading into arrhythmia, i.e., regional variability in contraction timing and repolarization that reflects underlying myocardial disorganization rather than kinematics alone. Mechanical dispersion, a speckle-tracking measure of the standard deviation of time-to-peak strain across left ventricular segments, was greater among aMVP patients (59 vs. 43 ms, *p* = 0.0002), independent of ejection fraction (*p* = 0.42), and predicted arrhythmic risk (OR 1.1 per ms increase; 95% CI 1.02–1.11; *p* = 0.006) [31]. Supporting this within an aMVP population, Vairo et al. [32] found that greater mechanical dispersion of the basal and mid-ventricular segments distinguished patients with major arrhythmic events requiring ICD implantation from those with high arrhythmic burden without major events, suggesting it may be a strong echocardiographic predictor of malignant arrhythmic events. In this sense, it functions less as a kinematic finding than as an imaging surrogate for the regional substrate that also manifests as inferobasal and papillary-muscle LGE on CMR and as inferolateral repolarization abnormalities on ECG. Together with abnormal regional mechanics, these features support a role for imaging-based phenotyping in arrhythmic risk stratification of aMVP.

### 2.3. aMVP Genetics and Systemic Vulnerability

Similar mechanical phenotypes among aMVP patients (MAD, bileaflet redundancy, and mechanical dispersion) can give rise to a wide variation of arrhythmic outcomes [6]. Genetic background, specifically cardiomyopathy gene variants, may thus shape proarrhythmic cardiac remodeling from chronic valvular strain.

Genome-wide association studies of over 4500 MVP patients have identified several possibly causal cardiomyopathy genes—*ALPK3*, *BAG3*, and *RBM20*—in MVP risk loci [33]. As further elucidated by Paldino et al. [34], *DSP*, *PKP2*, *LMNA*, and *FLNC* variants are potentially “arrhythmic genes” associated with a greater risk of SCD and malignant arrhythmias (*p* < 0.001) as compared to the traditionally recognized sarcomere and titin cardiomyopathy genes, independent of phenotypic classification. The two-hit model, genetic susceptibility paired with mechanical strain, suggests that malignant aMVP may arise when abnormal valvular mechanics act on an intrinsically vulnerable myocardium, increasing susceptibility to fibrosis, remodeling, and ventricular arrhythmia. This model further strengthens the observation that cfVE burden in aMVP can be disproportionate to the degree of mechanical stress [18], as genetic background may shape individual myocardial vulnerability. Although the mechanistic links between these candidate genes and arrhythmogenicity remain to be fully characterized [8,35], consideration of genetic determinants in aMVP may be valuable for evaluating arrhythmic predisposition, especially in the intermediate-risk subgroup.

### 2.4. From Mechanical Phenotype to Myocardial Disease

The clinical, imaging, and genetic features described above provide measurable anchors for translating the aMVP framework into candidate prevention endpoints. An arrhythmia burden endpoint from cfVE can be assessed by Holter monitoring, with added specificity when the extent of T-wave inversions is monitored [36]. Mechanical dispersion [31] and strain pattern endpoints [29], as measured by speckle-tracking echocardiography, can signify heterogeneity in ventricular contraction and its resulting impact on the inferobasal wall. Cardiac MRI endpoints indicating substrate progression include T1 mapping [36] and global extracellular volume (ECV%) [21] that detect diffuse interstitial fibrosis or edema, a likely precursor to irreversible replacement fibrosis. Finally, an intermediate endpoint of key significance for our population of interest is ^18^F-FDG uptake reduction as measured on hybrid PET/CMR. As a marker of subclinical myocardial inflammation observed in patients with bileaflet MVP and cfVE [13], this endpoint may help capture early and possibly reversible proarrhythmic substrate changes that can serve as a therapeutic target [37]. The colocalization [13] of inflammation and replacement fibrosis on LGE might serve as a further exploratory endpoint in tracking the evolution of the fibro-inflammatory signature in aMVP.

However, because the link between substrate modification and arrhythmia reduction has not been prospectively demonstrated in aMVP, the two should be treated as distinct endpoints rather than one as a surrogate for the other. A favorable substrate endpoint is supportive but neither necessary nor sufficient evidence of benefit, since trigger-directed therapies could reduce arrhythmia without altering imaging substrate. Trials should therefore pair the two: concurrent change would support the pathway, while divergence would indicate that the substrate measure does not capture it or that the intervention acts elsewhere.

### 2.5. Insights from Mitral Repair

Beyond localized traction imposed by leaflet billowing, MR itself contributes to myocardial remodeling and arrhythmogenesis [38]. Chronic primary MR drives left ventricular volume overload that promotes eccentric hypertrophy and interstitial and replacement fibrosis. LGE burden has also been shown to increase with MR severity, suggesting volume overload as an additional source of myocardial injury alongside mechanical stress [11]. The strength of association between MR and VA in primary MR remains constrained by limited prospective ambulatory rhythm monitoring and mechanistic adjudication of arrhythmic events [9]. Given that arrhythmic risk in aMVP is frequently disproportionate to MR severity, this suggests volume overload alone does not account for the regional fibro-inflammatory substrate we describe.

Given the dual contributions of volume overload and mechanical traction, mitral valve surgery may reduce papillary muscle and inferobasal mechanical stress, enabling favorable ventricular remodeling and theoretically reducing cfVE [39]. However, the current literature remains sparse and broadly suggests a mixed picture, with mitral valve surgery appearing to benefit younger patients (<60 years) or those experiencing implantable cardioverter–defibrillator (ICD) shocks [40,41]. This age disparity suggests mechanical unloading might be insufficient once an arrhythmic substrate has been solidified over time, and earlier interventions may be necessary for substrate reversal.

Importantly, diffuse interstitial fibrosis or edema, as measured by ECV% and iECV on CMR, was significantly reduced after surgical mitral valve repair, while replacement fibrosis on LGE remained unchanged [42]. These findings support early substrate reversibility through mechanical unloading, whereas LGE-defined replacement fibrosis appears less responsive over the observed follow-up interval. These findings have implications for therapeutic strategy, suggesting that early substrate-modifying interventions may offer benefit before LGE-defined replacement fibrosis is established, particularly in patients with active mechanical or diffuse fibrotic markers who have not yet developed overt malignant arrhythmia [16,21,43].

Notably, mitral intervention may act on ventricular arrhythmogenesis through two distinct mechanisms: relief of hemodynamically significant regurgitation and correction of abnormal valve–annulus mechanical interaction. A recent analysis by Sharew et al. comparing surgical repair and transcatheter edge-to-edge repair (TEER) among 1745 aMVP patients further supports this possibility [44]. In this analysis, aMVP was associated with worse five-year survival after TEER, whereas aMVP was not associated with worse survival after surgical repair [44]. Additionally, cfVE burden decreased after surgical repair, but was unchanged after TEER, and aMVP patients treated with TEER had higher rates of non-sustained ventricular tachycardia, ventricular tachycardia, and ICD implantation despite comparable MR reduction. While likely multifactorial, this difference highlights a broader mechanistic point: reducing MR may not necessarily correct the annular and ventricular mechanical abnormalities implicated in aMVP substrate formation, whereas annular stabilization from surgical approaches may be a more relevant mechanistic target [45,46]. These observations suggest future aMVP studies may test whether correcting the mechanical hit alters downstream substrate by measuring residual MR alongside variables such as MAD, annular dynamics, regional strain/mechanical dispersion, and ventricular arrhythmic burden, before and after intervention.

Coupled with surgical repair, pharmacologic substrate modification in the form of antifibrotic and anti-inflammatory approaches during this earlier phase may attenuate fibro-inflammatory and fibrotic remodeling that contributes to arrhythmogenic substrate formation. The evidence thus supports shifting the therapeutic focus from post-event device therapy to prevention focused on suppressing persistent inflammatory activity and modifying substrate before irreversible scar predominates.

### 2.6. Pathophysiologic Core: Mechanical Stretch to Fibro-Inflammatory Cascade in aMVP

**Figure 1:**
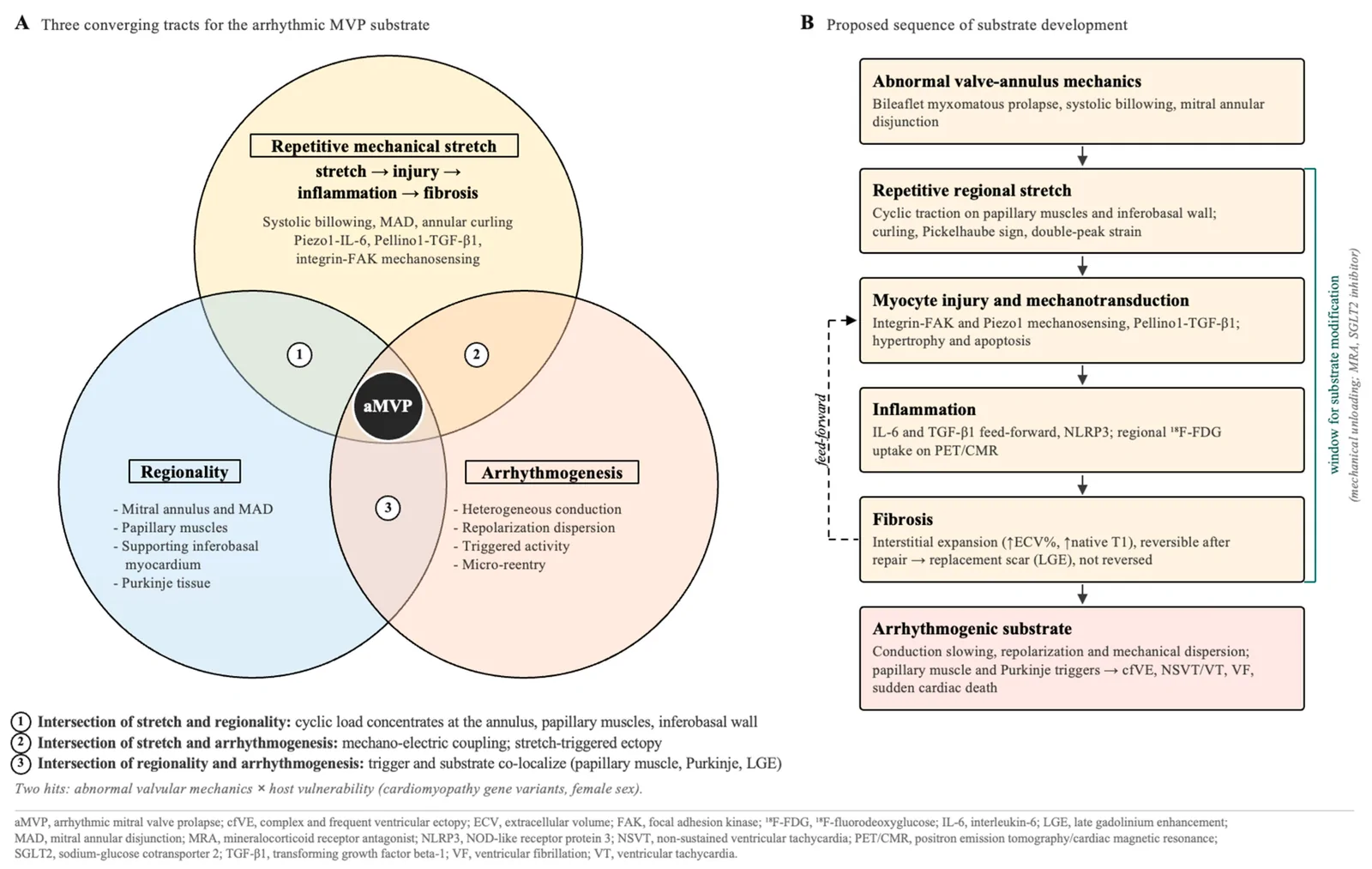
(**A**) Proposed pathophysiology of aMVP and (**B**) Proposed sequence of substrate development.

The pathogenesis of aMVP can be hypothesized as a two-hit model of cardiomyopathy, with hit one consisting of chronic mechanical stretch from systolic leaflet billowing and hit two consisting of the fibro-inflammatory cascade initiated by repetitive regional stress (Figure 1). Arrhythmia is considered the downstream consequence of these interactions between trigger, substrate, and inflammation as a modulator. Because the available aMVP data are largely cross-sectional, they cannot demonstrate a causal sequence between inflammation and fibrosis. The proposed direction is nonetheless consistent with established inflammatory–fibrotic pathways in other myocardial diseases and is supported by the anatomic colocalization of ^18^F-FDG uptake and late gadolinium enhancement.

### 2.7. Mechanotransduction: Molecular Links Between Stretch, Inflammation and Fibrosis

Cardiac fibroblasts detect mechanical stretch and resulting changes in myocardial stiffness through mechanosensors, driving fibrotic remodeling through several molecular signaling pathways [47]. Integrin-mediated mechanosensing has been well characterized: activation of integrin receptors by fibronectin and collagen results in focal adhesions with the extracellular matrix in response to changes in substrate stiffness [48]. The resulting fibrogenic cascades driven by tyrosine kinases (e.g., focal adhesion kinase) further drive pressure overload-induced myocardial fibrosis [49].

Piezo1 is a mechanosensitive cation channel that responds to pressure and stretch. Piezo1 induces calcium influx that stimulates interleukin-6 (IL-6) secretion, an inflammatory and profibrotic cytokine. As Blythe et al. [50] demonstrated, Piezo1 knockdown reduces IL-6 expression in cells cultured on soft collagen-coated substrates but not on regular tissue culture plates, providing evidence for the role of mechanosensing in the early detection of changes in substrate stiffness and regulation of inflammatory signaling. Of significance for aMVP pathogenesis, Piezo1 upregulation in cardiac fibroblasts promotes a positive feedback loop [51] leading to fibrosis progression and eventually electrical instability.

Downstream signaling cascades are recruited in the transmission of mechanical stretch, notably the Pellino1-mediated pathway that amplifies IL-6 production and TGF-β1 activation [52], which functions as a key regulator of fibrotic remodeling. Similar to Piezo1, a feed-forward loop is established: IL-6 induces myofibroblast activation via TGF-β1-mediated signaling [53]. IL-6 thus functions as a key driver of myocardial fibrosis and may serve as a potential therapeutic target. Galie et al. [54] provide further mechanistic insight into the connection between TGF-β1 signaling and angiotensin II type 1 receptor (AT1R), showing that interstitial fluid flow and cyclic mechanical strain exert opposing effects on cardiac fibroblasts in a delicate balance. Fluid flow appears to promote myofibroblast formation via coupled AT1R and TGF-β1 signaling, while cyclic strain inhibits it. Losartan treatment suppressed the flow-induced increase in myofibroblast activity, as did AT1R knockdown [54]. These findings may have applications in aMVP pathophysiology. Regions of altered hemodynamics in aMVP (e.g., altered flow patterns from MAD or fibrotic scarring in the strained inferobasal left ventricular wall) may promote AT1R-mediated myofibroblast activation and cardiac remodeling. Angiotensin II receptor blockade (e.g., losartan) of the flow-mediated phenotype in cardiac fibroblasts may therefore have antifibrotic potential in the treatment of aMVP. Together, these mechanotransduction pathways provide a molecular anchor for the stretch-to-inflammation model of aMVP.

Mechanotransduction may also involve post-transcriptional signaling through microRNAs and extracellular vesicle cargo, allowing mechanically stressed tissue to influence neighboring fibroblast and matrix behavior. Although this pathway has not been characterized in aMVP, it provides a plausible future direction for studying how regional stretch may propagate fibro-inflammatory remodeling.

### 2.8. Inflammation in aMVP: Contributions from Imaging

The prototypical aMVP phenotype diverges from the well-known understanding that MR severity dictates arrhythmic risk [38,55]. SCD has been reported predominantly among young to middle-aged women with minimal or mild MR, with some series reporting that females comprise an estimated 70–90% of affected patients [1,56]. Although likely multifactorial, this sex-based difference may be a starting point for further investigation. As measured by Tastet et al. and Chivulescu et al., diffuse interstitial fibrosis/edema was significantly elevated in female aMVP patients (*p* < 0.01) based on increased global ECV% measured by quantitative CMR [21] and lateral T1 (*p* = 0.004) [36], with adjustment for MR and replacement fibrosis by LGE in both studies. These findings indicate that diffuse interstitial fibrosis/edema compounds arrhythmic risk, with female sex as the common denominator.

Unlike focal replacement fibrosis, inflammation may represent a dynamic and potentially modifiable process. Surgical unloading data provide indirect support for this distinction, with reductions in diffuse interstitial fibrosis/edema but not LGE-defined replacement fibrosis after mitral repair [42]. This supports testing whether inflammatory and diffuse fibrotic molecular and imaging markers can be tracked and modulated before the emergence of fixed scar or malignant arrhythmia.

### 2.9. Fibrosis as the Arrhythmic Substrate: Diffuse Interstitial Disease and Focal LGE

Fibrosis provides one of the most consistent myocardial substrates linking aMVP to ventricular arrhythmia, but the available evidence does not require a single linear transition from diffuse interstitial fibrosis to focal replacement scar. Rather, diffuse fibrosis, focal scar, inflammatory activity, and electromechanical heterogeneity may coexist as distinct but interacting responses to abnormal regional mechanics. The detection of ventricular fibrosis in the setting of benign hemodynamics lays the groundwork for reconceiving both aMVP disease definition and risk stratification [20]. Catheter ablation studies have demonstrated abnormal tissue substrate characteristics at origin sites of VAs in aMVP [57], consistent with histopathological findings from autopsy [58] following SCD in MVP. In contrast, most aMVP cases with cfVE lack evidence of replacement scar by CMR [7,59]; rather, there is evidence of interstitial fibrosis correlating with VA and SCD risk [21]. This supports representing aMVP as a regional cardiomyopathy driven by mechanical stretch on the inferolateral left ventricular wall that induces fibro-inflammatory change, which may be an indicator of underlying arrhythmic susceptibility. The inclusion of interstitial fibrosis alongside focal replacement fibrosis on LGE in defining aMVP therefore might augment early electrical stratification [21,43] for at-risk patients lacking traditional features (e.g., severe MR or MAD) who have developed substrate that has not yet precipitated malignant VA, a group that appears enriched in women.

The crux of the localized mechanical injury model of aMVP is chronic maladaptive stretch, which is hypothesized to trigger myocardial hypertrophy and a regional inflammatory cascade [10,12]. This may drive reactive interstitial fibrosis, focal replacement scar, inflammatory activity, and electromechanical heterogeneity as distinct but interacting contributors to arrhythmogenic substrate. Interstitial fibrosis has been shown to slow action potential propagation and create conduction heterogeneity that drives ectopic automaticity [60]. The heterogeneous distribution of fibrosis in viable myocardium can enable breakthrough of micro-reentrant circuits, creating arrhythmogenic substrate [61].

Repetitive mechanical stretch from systolic leaflet billowing is hypothesized as the key precipitating factor in aMVP. The papillary muscles, mitral annulus, and left ventricular outflow tract have been identified as regions under high mechanical stress and are frequently the origin of VAs [19]. Recurrent stress strain subsequently activates myofibroblast transformation and the recruitment of inflammatory cells, a reactive process that can delay electrical conduction [62]. As discussed previously, hybrid PET/CMR studies [13] have demonstrated the coexistence of myocardial inflammation and replacement scar on LGE. Inflammation is of particular significance here as not only a potential link between diffuse interstitial fibrosis and eventual replacement fibrosis, but also as an active modulator of arrhythmogenesis. Given that approximately 25% of patients with MVP-related sudden cardiac death lack evidence of replacement fibrosis on LGE [12,63], this suggests the possible presence of inflammation as a mediator in arrhythmic substrate modeling. MVP animal models [14,64] have demonstrated regional myocardial fibrosis in the basal inferolateral wall and papillary muscles, paralleling fibrotic changes observed in humans. Ventricular fibrosis has been associated with adverse outcomes. Figliozzi et al. showed that LGE presence was associated with adverse outcomes in 474 MVP patients without severe MR or left ventricular dysfunction (HR 4.2; 95% CI 1.5–11.9; *p* = 0.006). LGE extent was also associated with outcome (HR 1.2 per 1% increase; 95% CI 1.1–1.4; *p* = 0.006), and advanced age and severe MR were associated with increased fibrosis [65]. In a recent meta-analysis by Gatti et al. [66], LGE showed promise as a CMR marker with strong predictive value for identifying MVP patients at risk for complex VAs (log OR 2.12, 95% CI 1.00–3.23).

Catheter ablation has been important not only therapeutically, but also mechanistically, by localizing the sites of ectopy and malignant ventricular arrhythmia in aMVP. Mapping studies have identified papillary muscle and Purkinje-related triggers [67], including VF-triggering ectopy [57], and have helped define VT substrates in regions exposed to abnormal valvular myocardial mechanics. These findings allow the electrophysiologic phenotype of aMVP to be interpreted alongside imaging and pathologic observations, linking arrhythmic triggers and substrate to regions of known stretch, injury, inflammatory activity, and fibrosis. Catheter ablation for MVP-related cfVE has limited durability, with recurrence rates estimated up to 42% at 1.3 years [7,9,57], with possible reasons being incomplete ablation of deep intramural sites of origin, the presence of multiple arrhythmogenic foci, and substrate progression in the setting of ongoing mechanical stress [7,45,68].

## 3. Part II: Substrate-Modifying Treatments

A prevention-grade approach to aMVP may involve upstream pharmacotherapy intervention targeting the mechanotransduction–inflammation–fibrosis sequence that induces an arrhythmogenic substrate. Within this conceptual framework, we review potentially repurposable substrate-modifying therapies on four axes: interstitial and replacement fibrosis, innate immunity and inflammasome signaling, mechanosensing and neurohormonal activation, and calcium modulation. We also evaluate these candidates by several primary criteria: mechanistic fit (alignment with pathophysiologic pathways described above), human cardiac evidence, safety, and feasibility in prevention trials (Table 1).

### 3.1. Mineralocorticoid Receptor Antagonists

Mineralocorticoid receptor antagonists (MRAs, spironolactone, eplerenone, and finerenone) block aldosterone/mineralocorticoid receptors in the distal nephron, exerting mild diuretic effects by decreasing sodium reabsorption and increasing potassium and hydrogen excretion [72]. MR antagonism upregulates antifibrotic genes and suppresses the expression of profibrotic genes and plasminogen activator inhibitor-1 [95], core components of the mechanotransduction pathways described earlier. Moreover, MR activation can impact electrical remodeling [69] through modulation of sarcoplasmic reticulum calcium flux and action potential duration, ultimately promoting rhythm disorders [70]. MR antagonism therefore may have short-term antiarrhythmic and long-term antifibrotic applications. RALES [96] and EPHESUS [97] underscored the roles of MRAs in decreasing the incidence of SCD in post-MI and NYHA III-IV heart failure, likely by reducing malignant VAs. RALES and EPHESUS enrolled post-MI and heart failure populations. Extrapolating to aMVP, these findings may have direct mechanistic application to the use of MRAs in aMVP, given the overlap between deleterious arrhythmogenic remodeling in MVP and heart failure. However, a retrospective study by Schupp et al. [73]. among 366 ICD recipients with systolic heart failure showed that MRA therapy was not associated with a first recurrence of malignant VA at a longer five-year follow-up (HR 1.067; 95% CI 0.736–1.546; *p* = 0.732), suggesting a differential impact of MRAs on substrate maturity and the need for further studies to conclusively determine the antiarrhythmic impact of MRA therapy.

In addition to antiarrhythmic benefit, MRAs might have anti-inflammatory and antifibrotic potential. In MAGMA [74], spironolactone dosed at 25 mg daily for one year significantly reduced native T1 values (−10.1 ms spironolactone group vs. 26.0 ms placebo group, *p* = 6.33 × 10^−4^) with parallel regression of left ventricular mass (*p* = 0.001) independent of ambulatory blood pressure. In HOMAGE [98], spironolactone significantly reduced markers of type 1 collagen synthesis (mean difference −8.1; 95% CI −11.9 to −4.3 μg/L; *p* < 0.0001), providing further human cardiac evidence for both the anti-inflammatory and early antifibrotic therapeutic properties of MRAs. Among dilated cardiomyopathy patients, spironolactone has also reduced the extent of collagen deposition with concomitant regression in myocardial fibrosis, notably among patients with the greatest baseline fibrotic burden [75]. Spironolactone at a daily dose of 25–50 mg is a cost-effective steroidal MRA, backed by robust human evidence for myocardial fibrosis reduction [76]. The nonsteroidal MRA finerenone may have antifibrotic advantages and fewer antiandrogenic effects than spironolactone, although its role in aMVP remains untested [99].

### 3.2. Sodium–Glucose Cotransporter 2 Inhibitors

Sodium–glucose cotransporter 2 (SGLT2) inhibitors (canagliflozin, dapagliflozin, empagliflozin) inhibit glucose reabsorption within the proximal renal tubules, resulting in glucosuria and blood pressure reduction [77]. SGLT2 inhibitors are mechanistically attractive in aMVP because they may affect several components of the proposed disease biology, including inflammatory signaling, myocardial fibrosis, adverse remodeling, and calcium handling. Using experimental models of heart failure, Byrne et al. [78] demonstrated that SGLT2 inhibitors attenuate the activation of the inflammasome NLRP3, directly targeting the immune signaling cascades involved in inflammatory mechanotransduction. In a randomized trial of canagliflozin versus sitagliptin in high cardiovascular risk type 2 diabetes mellitus [100], canagliflozin was associated with an adjusted between-group difference in extracellular volume change on CMR of −3.67% (95% CI −5.33 to −2.01; *p* < 0.001), providing imaging evidence of antifibrotic benefit. Similarly, a randomized trial of dapagliflozin in HFpEF with type 2 diabetes [79] showed that dapagliflozin significantly reduced myocardial fibrosis (mean ΔECV: −3.5% vs. −0.8% with placebo; *p* < 0.001), with a reduction in left ventricular mass index and improved functional capacity. The mechanistic profile of SGLT2 inhibitors suggests these agents can target the key components of the pathogenic cascade in aMVP, including hypertrophic and fibrotic substrate in addition to modulation of inflammation.

Beyond fibrosis, SGLT2 inhibitors further demonstrate evidence of potential antiarrhythmic and sudden-death benefit. Through inhibition of NHE1 (late inward sodium current) and calcium–calmodulin-dependent protein kinase II (CaMKII), SGLT2 inhibitors modulate calcium flux and thus arrhythmogenicity [80]. A SMART-C meta-analysis [81] of 78,607 patients across 11 trials showed SGLT2 inhibitors significantly reduced SCD (HR 0.86; 95% CI 0.78–0.95). In DAPA-HF [101], dapagliflozin reduced the risk of any VAs, resuscitated cardiac arrest, or sudden death by 21% when it was added to other agents previously shown to reduce VAs (e.g., beta-blockers, MRAs) (HR 0.79; 95% CI 0.63–0.99; *p* = 0.037). Antiarrhythmic and sudden death benefit was further supported in a subsequent pooled analysis [102] of DAPA-HF and DELIVER (11,007 patients across the heart failure disease spectrum), which found that dapagliflozin reduced cardiovascular death (HR 0.86, 95% CI 0.75–0.98; *p* = 0.02) with a nonsignificant trend toward lower sudden death risk (HR 0.84; 95% CI 0.70–1.01; *p* = 0.07). Of note, these data derive from heart failure and high-risk diabetic populations, and relevance to aMVP is inferential. These reductions in SCD among heart failure patients, who possess an established substrate, suggest SGLT2 inhibitors may play a role in slowing or even suppressing the precipitation of arrhythmia from the substrate, which is the goal of pharmacotherapy in intermediate-risk aMVP patients.

### 3.3. Renin–Angiotensin–Aldosterone System Blockade

Renin–angiotensin–aldosterone system (RAAS) blockade is less directly supported in aMVP than MRAs or SGLT2 inhibitors but remains relevant because angiotensin II and aldosterone signaling intersect with fibroblast activation, extracellular matrix remodeling, and electrical instability. There are four major classes of RAAS blockade: ACE inhibitors, angiotensin receptor blockers (ARBs), angiotensin–receptor neprilysin inhibitors (ARNIs), and direct renin inhibitors [103]. RAAS activation promotes proarrhythmic structural and electrical remodeling. ARNIs (sacubitril/valsartan) have been shown to promote reverse remodeling [104] and mitigate myocardial fibrosis [105], suggesting protective benefit for substrate progression to malignant VAs. While RAAS blockade has shown promising benefit in heart failure [83,106], it may have mixed effects on aMVP pathophysiology. Strauss et al. [82] observed that ACE inhibitors and ARBs decreased regurgitant fraction in MVP by 8.1% (95% CI 4.3–11.9; *p* < 0.05). However, Kizilbash et al. [71] demonstrated that vasodilators may decrease left ventricular size and mitral closing force, thus worsening MVP and MR.

Emerging evidence further suggests that RAAS signaling may intersect with post-transcriptional regulation of myocardial remodeling through microRNA pathways. Although these pathways have not yet been specifically characterized in aMVP, microRNA-mediated regulation of fibroblast activation, extracellular matrix turnover, and TGF-β signaling may represent an additional mechanism through which RAAS inhibition could exert substrate-modifying effects beyond hemodynamic unloading alone.

### 3.4. Anti-Inflammatory Agents

#### 3.4.1. Colchicine

Colchicine demonstrates anti-inflammatory effects by inhibiting β-tubulin polymerization into microtubules [107]. A short course of colchicine in the intermediate-risk population may therefore limit progression to fibrosis. We specifically evaluate the targeted anti-inflammatory therapies colchicine and IL-1 inhibition as substrate-modifying agents [85].

Colchicine has been shown to suppress activation of the NLRP3 inflammasome with overall attenuation of IL-6 and CRP concentrations [84], directly targeting the immune signaling pathways underlying aMVP pathogenesis. Colchicine directly suppresses myofibroblast proliferation through TGF-mediated fibrosis pathways, reducing collagen synthesis and deposition [86]. It also has strong human cardiac evidence: LoDoCo2 [87] demonstrated that colchicine 0.5 mg daily reduced a composite primary endpoint of cardiovascular death, spontaneous MI, ischemic stroke, and ischemia-driven coronary revascularization (HR 0.69; 95% CI 0.57–0.83; *p* < 0.001). We note, however, that these trials examined only atherosclerotic and post-MI populations; thus, relevance to aMVP is an extrapolation. But as a generic, cost-effective, and orally administered drug, colchicine might be a practical consideration for prevention trials with intermediate-risk cohorts. The safety profile of colchicine is well established from robust cardiovascular trials such as LoDoCo2 [87] and COLCOT [88], with no routine laboratory monitoring required.

#### 3.4.2. IL-1 Blockade

IL-1 blockade prevents IL-1α/β from activating IL-1 receptors, thus reducing fever, vasodilation, and inflammatory cytokine signaling. There are three main agent classes: receptor antagonist (anakinra), decoy receptor fusion protein (rilonacept), and neutralizing antibody (canakinumab) [89]. IL-1 blockade with anakinra or canakinumab has been shown to directly target the NLRP3–IL-1β–IL-6–CRP axis underlying mechanotransduction-driven inflammation in aMVP [108]. Similar to colchicine, IL-1 blockade is supported by extensive human cardiac evidence. In CANTOS [90,91], canakinumab reduced recurrent vascular events by 15–17% in stable statin-treated patients with elevated hs-CRP over a median of 3.7 years, with a recent exploratory analysis [92] indicating dose-dependent reduction in heart failure hospitalization and mortality. In VCU-ART2 [93], anakinra reduced hs-CRP levels alongside a significant reduction in new-onset heart failure after STEMI (5% vs. 30%; *p* = 0.035), demonstrating anti-inflammatory properties. As noted above, these trials also examined atherosclerotic/post-MI populations with no aMVP-direct data.

Given that similar inflammatory pathways are implicated in aMVP, IL-1 blockade may potentially disrupt the fibro-inflammatory cascade in aMVP, particularly enriched in the intermediate-risk subgroup of patients with imaging-proven inflammation but without replacement scar. For proof-of-concept trials, anakinra may be a superior candidate to canakinumab given dual blockade of IL-1α and IL-1β and a shorter half-life (four to six hours vs. 26 days), allowing rapid washout [108]. However, IL-1 blockade is limited by cost, injection-based delivery, and infection risk [108], making it better suited to small proof-of-mechanism studies than broad prevention trials.

### 3.5. Pirfenidone: An Imaging Endpoint Antifibrotic Prototype

Pirfenidone, an oral hemodynamically neutral antifibrotic agent for idiopathic pulmonary fibrosis, is a potential candidate for use in proof-of-concept clinical trials to evaluate the hypothesis that myocardial fibrosis in aMVP is pharmacologically modifiable. Pirfenidone reduces the expression of proinflammatory cytokines TNF-α, IL-1β, and IL-4 [109]. It additionally targets the TGF-β1/Smad3 signaling pathway and suppresses myofibroblast transformation [110], core components of the mechanotransduction-to-fibrosis pathway implicated in aMVP.

PIROUETTE [111] provided the strongest human proof-of-concept for pirfenidone to date. Pirfenidone treatment over 52 weeks reduced the primary endpoint of myocardial ECV by 1.21% compared to placebo (95% CI −2.12 to −0.31; *p* = 0.009) in patients with HFpEF and CMR-quantified myocardial fibrosis (ECV ≥ 27%). Pirfenidone also appears to have a favorable safety profile, with adverse effects comparable to placebo, commonly nausea, insomnia, and rash. PIROUETTE provided a starting template for implementation of imaging endpoints to depict pharmacologic regression of cardiac fibrosis in aMVP. The enriched imaging strategy (ECV ≥ 27%) for enrollment demonstrates that substrate-driven patient selection is feasible and likely advantageous for later signal detection. The effect size (1.21% ECV reduction) further provides a starting foundation for future longitudinal aMVP prevention trials, with a focus on assessing the magnitude of fibrosis regression that results in a significant reduction in malignant VA burden. Being hemodynamically neutral, pirfenidone’s antifibrotic impact may be isolated from other hemodynamic confounders and thus may confer a strong mechanistic advantage for assessing the link between dose-dependent pharmacologic substrate modification and arrhythmic outcomes [112].

Its practical limitations include gastrointestinal intolerance, photosensitivity, liver enzyme monitoring, cost, and limited cardiac experience, making it more useful as a mechanistic imaging endpoint prototype than as a near-term clinical therapy [109,112].

### 3.6. Emerging Antifibrotic Agents

Several additional pharmacologic agents show promising preclinical evidence for substrate modification but lack the compelling clinical background of the agents described above. However, they do merit further discussion and investigation as potential therapeutic candidates that may provide further insight into the localized mechanical injury model of aMVP.

#### 3.6.1. Galectin-3 Inhibitors

Galectin-3 is a lectin protein at the intersection of cardiac remodeling and inflammation modulation. Shah et al. [113] demonstrated a positive correlation between galectin-3 levels and echocardiographic markers of ventricular dysfunction and MR/TR severity, suggesting applicability to aMVP. Inhibition of galectin-3 has also been shown to reduce fibrotic progression in post-MI patients, with Wang et al. [114]. showing greater therapeutic efficacy than losartan and spironolactone monotherapy. However, human cardiac data on galectin-3 use are limited to observational studies, and clinical translation remains at an early stage.

#### 3.6.2. MicroRNAs (miRNAs)

miRNAs are small, noncoding RNAs transcribed in the nucleus that regulate target mRNA molecules through degradation or translational repression [94]. Notably, a single miRNA can regulate multiple mRNA molecules, with individual mRNA molecules possessing multiple miRNA binding sites, positioning these molecules as potential therapeutic targets in ECM remodeling and electrophysiologic regulation [115]. Targeting miRNAs, including miR-29a and miR-133a, has been proposed to attenuate maladaptive cardiac structural remodeling [116]. miR-133a has been identified as a negative regulator of CTGF expression and fibroblast activation, with experimental evidence supporting antifibrotic effects in mechanically stressed cardiovascular tissues. Although much of this evidence derives from vascular rather than myocardial models, it supports the broader concept that mechanically induced fibro-inflammatory remodeling may be modifiable through post-transcriptional regulatory pathways. However, effective delivery of miRNA-based therapeutics remains a challenge to overcome before clinical translation. Given that circulating miRNAs are relatively stable in plasma and can reflect underlying pathophysiology, miRNAs may further serve as diagnostic or prognostic biomarkers [117].

#### 3.6.3. Connective Tissue Growth Factor (CTGF) Inhibitors

CTGF promotes fibroblast proliferation and extracellular matrix production. CTGF blockade has been proposed as an approach to suppress profibrotic pathways [118]. However, cardiac applications of CTGF monoclonal antibodies have not yet been studied.

## 4. Part III: Translational Applications and Limitations

### 4.1. Animal and Ex Vivo Platforms

The fibro-inflammatory remodeling signature of aMVP requires preclinical support before initiation of clinical trials. Animal and ex vivo models may provide further insight into the coupled valve–ventricle disease model of aMVP. The *Dzip1^S14R/^*^+^ murine model [14] of MVP demonstrated progressive left ventricular fibrosis in the basal inferolateral wall and papillary muscles, providing further evidence for the dynamic effects of valve pathology and myocardial remodeling. Large animal models [119] (e.g., swine and sheep) can further characterize valvular-myocardial biomechanics, providing an advantage over ex vivo bio-simulators with the use of FDG-PET, strain imaging, and electroanatomic mapping techniques [9]. iPSC-derived cardiac tissues expressing fibroblast markers have demonstrated mechanotransduction-driven fibrotic remodeling, with antifibrotic agents (e.g., pirfenidone, hepatocyte growth factor) shown to suppress fibrotic changes in vitro [120]. Goldfracht et al. [121] Showed that engineered cardiac tissue models from hiPSC-derived cardiomyocytes and cardiac ECM can model reentrant arrhythmias, while Hegyi et al.’s [122] patch-clamp-in-gel platform models the path from mechanical loading to arrhythmogenesis. Both animal models and ex vivo platforms can provide further translational proof-of-concept support, while the clinical cohorts described above can mature over time.

### 4.2. Digital Twins

Patient-specific computational models, or digital twins, offer a promising digital representation of valve–ventricle biomechanics and enable prediction of fibrosis-induced arrhythmogenesis. Digital twins can integrate patient-specific cardiac motion (annular dynamics, bileaflet motion) and patient-specific fibrosis distribution [123]. Although simulations of fibrotic remodeling have largely been in the context of atrial fibrillation [124], mechanistic parallels to fibrotic changes and arrhythmia inducibility in aMVP can be drawn. As Trayanova et al. [125] described, multiscale computational models can predict abnormal automaticity in the myocardial substrate, an approach already validated in ischemic (ICM) and nonischemic cardiomyopathy (NICM)-related arrhythmogenesis.

Such models hold promising applications for enrichment optimization and endpoint selection. Virtual cohorts can serve as a preliminary template for refining enrollment by testing which mechanical features (e.g., MAD, MR) most strongly impact substrate advancement [126] and identifying the optimal imaging approaches to track intervention effects. Patient-specific models may further enable stratification into subgroups using mechanical–electrophysiological coupling, with Serra et al. [127]. showing benefits in arrhythmic risk stratification. Digital twins are a valuable resource to translate the two-hit hypothesis of aMVP into quantitative simulation, allowing for multifactorial testing before clinical trial initiation.

### 4.3. Anticipated Limitations

Here we address the major criticisms of the pharmacotherapeutic approach to aMVP and its underlying biology. MVP encompasses a heterogeneous spectrum, from isolated single leaflet prolapse to diffuse myxomatous disease with extensive cardiac remodeling. Familial and genetic studies have demonstrated wide-ranging phenotype variability, with critics suggesting this complex disease spectrum precludes a consolidated treatment approach [128]. However, we emphasize an enrichment strategy with selection criteria specific to patients with mechanical and myocardial features suggestive of substrate presence. Heterogeneity in aMVP presentation encourages further precision phenotyping, rather than abandonment of therapeutic treatment.

We also recognize the case for causal ambiguity in the role of fibrosis as a driver or bystander in pathogenesis, questioning the utility of antifibrotic approaches. However, multiple analyses have supported fibrosis as a substrate for eventual arrhythmogenesis. The meta-analysis by Disertori et al. [129] revealed a dose–response effect between ventricular fibrosis extent and arrhythmic risk. In both ICM and NICM over a median follow-up of 5.3 years, both the presence and extent of fibrosis independently predicted arrhythmic risk (ICM aHR 4.61; 95% CI 2.75–7.74; *p* < 0.001 and NICM aHR 1.10; 95% CI 1.05–1.16 at 1% LGE increase; *p* < 0.001). Conversely, a subset of MVP-related sudden death occurs without MRI-detectable replacement fibrosis [12,63], indicating that fibrosis may in part be a marker rather than the sole driver, or that this finding may reflect limitations in our ability to accurately identify and quantify it on imaging. Overall, fibrosis as an arrhythmogenic substrate is among the most firmly established paradigms in cardiac electrophysiology, and targeted ablation of substrate within scar can demonstrably terminate the corresponding arrhythmia, including at MVP-related scar and Purkinje trigger sites. Judged against Bradford Hill considerations, the sole unmet criterion is experimental confirmation within aMVP, which is the reason we propose future prevention cohorts and trial designs. An analogous caution applies to the inflammatory component of the proposed cascade: diffuse interstitial signal and regional FDG uptake are compatible with several overlapping mechanistic roles, and their causal position in the arrhythmic cascade remains unproven in aMVP.

Safety and long-term tolerability are further considerations in prevention cohorts, given that measurable substrate regression may occur over years. The pharmacologic agents prioritized for implementation (MRAs, SGLT2 inhibitors, RAAS blockade) are backed by strong safety profiles from application within and beyond heart failure cohorts. However, trial design should integrate run-in periods to further assess tolerability before randomization and refine monitoring protocols.

## 5. Conclusions and Future Directions

We present a narrative synthesis of contemporary literature suggesting that aMVP may represent a regional, stretch-induced cardiomyopathy with a characteristic fibro-inflammatory signature. This synthesis encourages a transition from the reactive device-driven approach to biologically informed prevention therapy. The evolution of heart failure management offers a template for a paradigm shift in the clinical approach to aMVP from rescue to remodeling therapy. The CONSENSUS trial, which demonstrated that pharmacotherapy could modify disease progression, set the foundation for the subsequent neurohormonal blockade that enabled a shift from palliation to disease modification with demonstrated mortality reduction. aMVP remains at the pre-CONSENSUS stage, with a strong case for future prevention trials that can drive a shift towards arrhythmic substrate modification. We emphasize focusing on the intermediate-risk subgroup of patients, with a substrate that has not yet precipitated malignant VA, as a population particularly amenable to substrate-modifying therapy before the development of irreversible replacement fibrosis.

While the evidence base continues to evolve, biologically informed prevention trials in aMVP are supported by a measurable trajectory of the fibro-inflammatory cascade, mechanistic nodes targetable by repurposable agents (e.g., MRAs, SGLT2i, RAAS blockade, colchicine) in mechanistically adjacent populations, and surgical insights from mitral repair. If at least one therapy class can quantifiably move substrate endpoints, event-driven prevention trials are a reasonable next step. The goal of treatment is not to eliminate ectopic burden, but rather to slow or reverse the fibro-inflammatory trajectory that transforms valvular dysfunction into a malignant arrhythmic substrate in a key subset of patients. This framework highlights several plausible mechanistic nodes for future study, including mineralocorticoid signaling, SGLT2-linked inflammatory and remodeling pathways, AT1R signaling, and IL-1/IL-6-mediated inflammation. Next steps may include phase 2 trials with enriched cohorts and imaging-driven substrate endpoints over measurable durations, supported by preclinical animal models and digital twins integration. Reconceiving aMVP as a regional stretch-induced cardiomyopathy may provide a framework for moving from rescue therapy toward biologically informed prevention.

## Figures and Tables

**Table 1:** Summary of Candidate Substrate-Modifying Therapies.

Drug Class	Mechanistic Target	Human Cardiac Evidence	Evidence Category (A–D)	Relevance to aMVP	Safety/Feasibility	Prioritization for Future Study
**MRAs** (spironolactone, eplerenone, finerenone)	MR-mediated fibroblast activation, TGF-β1/PAI-1 expression, macrophage profibrotic signaling, mild diuretic effect by decreasing sodium reabsorption and potassium/hydrogen excretion [66].	MAGMA [69] trial: spironolactone reduced native T1 by 10.1 milliseconds over 12 months (*p* = 0.0006); HOMAGE [70] trial: reduced collagen synthesis markers; HFpEF data: slowed ECV expansion over 6 months.	C (heart failure, post-myocardial infarction, chronic kidney disease)	Targets the mechanotransduction-to-fibrosis cascade at multiple nodes; RALES [71]/EPHESUS [72] demonstrated sudden cardiac death reduction; anti-inflammatory and antifibrotic potential.	Generic and inexpensive; requires potassium/creatinine monitoring; gynecomastia risk with spironolactone (10–15%); finerenone avoids antiandrogenic effects but has CYP3A4 interactions [66,73].	**Highest:** MAGMA [69] provides a direct trial template (12-month CMR-based fibrosis endpoints) with established monitoring protocols.
**SGLT2 inhibitors** (dapagliflozin, empagliflozin, canagliflozin)	NLRP3 inflammasome suppression, macrophage polarization, and myofibroblast inhibition via SIRT6 and NHE1 inhibition [74].	Canagliflozin is associated with an adjusted between-group difference in ECV change of −3.67 percentage points over 26 weeks (*p* < 0.001) [75] compared to sitagliptin; dapagliflozin reduced myocardial fibrosis by 3.5% vs. 0.8% placebo over 12 months in HFpEF (*p* < 0.001) [76].	C (heart failure, type 2 diabetes mellitus)	Multi-target relevance across inflammation, fibrosis, and ion channel pathways; SMART-C collaborative meta-analysis: significant sudden cardiac death reduction (HR 0.86) [77]; DAPA-HF: 21% reduction in ventricular arrhythmia/sudden cardiac death composite (HR 0.79) [78].	Favorable tolerability; no electrolyte monitoring required; genital mycotic infection (10–15%); rare diabetic ketoacidosis; protective against acute kidney injury; minimal drug interactions.	**High:** Strongest near-term feasibility: broad cardiovascular experience, favorable tolerability, minimal monitoring burden, and supportive imaging-endpoint data.
**RAAS blockade** (ACE inhibitors, ARBs, ARNi)	AT1R-mediated neurohormonal activation, TGF-β/Smad2/3 signaling in valvular interstitial cells [79].	ARNIs (sacubitril/valsartan) have been shown to promote reverse remodeling [80] and mitigate myocardial fibrosis [81]. Losartan attenuates TGF-β signaling and extracellular matrix production in human myxomatous valve tissue [51].	B+ C (heart failure, MVP-direct regurgitant fraction data [71,82])	Targets both the mechanical stressor (valve remodeling) and the myocardial response (fibrosis); potential platform therapy to reduce noise across trial arms.	Well-established long-term safety profile; hypotension and renal monitoring required; widely available and generic.	**Moderate:** Strong mechanistic rationale for valve–ventricle coupling; less direct evidence for myocardial fibrosis regression than MRAs/SGLT2 inhibitors; well-positioned as background therapy in combination trials.
**Colchicine**	Microtubule disruption, NLRP3 inflammasome inhibition, neutrophil chemotaxis suppression, IL-1β/IL-6 downstream reduction [83].	COLCOT [84] and LoDoCo2 [85] demonstrated cardiovascular event reduction in atherosclerotic disease; no direct myocardial fibrosis imaging data in non-ischemic populations.	C (atherosclerosis, post-myocardial infarction)	Targets the inflammatory activation phase of the arrhythmic mitral valve prolapse cascade; cost-effective; could be tested as short-course therapy in patients with imaging-confirmed inflammation.	Inexpensive, generic; GI side effects (diarrhea) dose-limiting; narrow therapeutic index; requires renal/hepatic dose adjustment; CYP3A4 interactions.	**Moderate:** Practical for proof-of-concept inflammation-targeted trials; clear imaging (FDG-PET) and biomarker (hs-CRP, IL-6) endpoints.
**IL-1 pathway blockade** (anakinra, canakinumab)	Direct IL-1β neutralization (canakinumab) or IL-1 receptor antagonism (anakinra); upstream suppression of IL-6, hs-CRP, and downstream inflammatory cascades [86,87].	CANTOS [88,89]: canakinumab reduced cardiovascular events by 15–17% among statin-treated patients; VCU-ART2 [90]: anakinra reduced inflammation and new-onset heart failure after STEMI^.^	C (atherosclerosis, post-myocardial infarction)	Targets the inflammatory signaling connecting mechanotransduction and fibrosis; may be most relevant in the subset with proven active inflammation on hybrid imaging.	Injection-based delivery; infection risk; high cost (canakinumab); anakinra requires daily injection; feasibility barriers for long-term prevention [90].	**Lower.** Best suited as mechanistic proof-of-concept in small cohorts with documented inflammation; cost and delivery route limit scalability to trials.
**Pirfenidone**	Inhibits TGF-β–mediated collagen synthesis, fibroblast proliferation, and myofibroblast differentiation [91,92].	PIROUETTE trial [93] (n = 47): pirfenidone reduced myocardial ECV by 1.21% vs. placebo over 52 weeks (*p* = 0.009) in HFpEF. The first randomized controlled trial demonstrating pharmacologic myocardial fibrosis regression by CMR.	C (HFpEF)	Proof-of-principle that imaging-driven substrate regression is achievable; validates ECV as a modifiable endpoint.	GI side effects (nausea, anorexia) and photosensitivity common; hepatotoxicity monitoring required; tolerability may limit long-term use in prevention cohort.	**Moderate** (as mechanistic tool). PIROUETTE provides a mechanistic imaging-endpoint prototype for substrate regression, but not yet a near-term clinical therapy.
**Emerging targets** (galectin-3 inhibitors)	Fibroblast activation and collagen deposition.	Positive correlation between galectin-3 levels and imaging evidence of ventricular dysfunction, suggesting possible application to arrhythmic mitral valve prolapse [94].	D (preclinical/observational)	May serve as enrichment biomarkers or pharmacodynamic endpoints.	Early phase; safety profiles incompletely characterized; most lack oral formulations or established dosing.	**Lowest.**

Evidence category (see compact classification table): A = direct human aMVP evidence (arrhythmia-selected MVP populations); B = human MVP evidence without specific arrhythmic selection; C = indirect evidence from other cardiovascular populations (heart failure, post-myocardial infarction, atherosclerosis); D = preclinical evidence (animal, ex vivo, in vitro). No agent currently has Category A treatment evidence in aMVP. ACE inhibitors: angiotensin-converting enzyme inhibitors. ARBs: angiotensin II receptor blockers. ARNi: angiotensin-receptor neprilysin inhibitors. AT1R: Angiotensin II type 1 receptor. CANTOS Trial: Canakinumab Anti-Inflammatory Thrombosis Outcomes Study. CMR: Cardiac magnetic resonance. COLCOT Trial: Colchicine Cardiovascular Outcomes Trial. CYP3A4: Cytochrome P450 3A4. DAPA-HF Trial: Dapagliflozin and Prevention of Adverse Outcomes in Heart Failure. ECV: Extracellular volume. EPHESUS Trial: Eplerenone Post-Acute Myocardial Infarction Heart Failure Efficacy and Survival Study. FDG-PET: Fluorodeoxyglucose Positron Emission Tomography. HFpEF: Heart failure with preserved ejection fraction. HOMAGE Trial: The Heart “Omics” in AGEing Randomized Clinical Trial. hs-CRP: High-sensitivity C-reactive protein. IL: Interleukin. LoDoCo2 Trial: Low Dose Colchicine for Secondary Prevention of Cardiovascular Disease 2. MAGMA Trial: Mineralocorticoid Receptor Antagonism Clinical Evaluation in Atherosclerosis. MRAs: Mineralocorticoid receptor antagonists. NHE1: Sodium-hydrogen antiporter 1. NLRP3: NOD-like receptor protein-3. PAI-1: Plasminogen activator inhibitor-1. RAAS blockade: Renin–angiotensin–aldosterone system blockade. RALES trial: Randomized Aldactone Evaluation Study. SGLT2: Sodium–glucose cotransporter 2. SIRT6: Sirtuin 6. STEMI: ST-elevation myocardial infarction. TGF-β1: Transforming growth factor beta-1. VCU-ART2 Trial: Virginia Commonwealth University-Anakinra Remodeling Trial 2.

## Data Availability

No new data were created or analyzed in this study. Data sharing is not applicable to this article.
